# Vaginal microbiome distinction in women with HPV+, cervical intraepithelial neoplasia, and cervical cancer, a retrospective study

**DOI:** 10.3389/fcimb.2024.1483544

**Published:** 2025-01-17

**Authors:** Yuanyue Li, Xiaomei Wu

**Affiliations:** ^1^ Department of Gynecology, The First People's Hospital of Yunnan Province, Kunming, China; ^2^ The Affiliated Hospital of Kunming University of Science and Technology, Kunming, Yunnan, China

**Keywords:** human papillomavirus, HPV genotypes, vaginal microbiome, cervical intraepithelial neoplasia, cervical cancer

## Abstract

**Introduction:**

The vaginal microbiota is a complex and dynamic micro-ecosystem that plays a pivotal role in protecting the host from various pathogens. Previous studies have investigated the diversity of the vaginal microbiome and its association with health outcomes, particularly the development of HPV-related disorders. This study aimed to investigate the correlation between the vaginal microbiota, HPV infection, cervical intraepithelial neoplasias (CINs), and cervical cancers in 69 women.

**Methods:**

DNA was extracted from vaginal samples, followed by HPV genotyping through PCR and sequenced of the16S rRNA gene.

**Results:**

Our results revealed that *Lactobacillus* was the predominant bacterium across all groups, with prevalence rates of 60.2% in women with HPV+, 63.9% in CINI, 97.7% in CINII, 52.0% in CINIII, 36.9% in cervical cancer, and 70.9% in NILM (normal cytology). Additionally, an elevated proportion of Gardnerella was identified as a high-risk bacterium associated with HPV infection, potentially contributing to the progression of cervical lesions. High-risk HPV genotypes, particularly HPV16, 52, and 33, were found to be more prevalent among women with HPV+, CIN, and cervical cancer. We also observed significantly higher alpha diversity in the vaginal microbiome of women with HPV+ and CIN, as indicated by increased Sobs, Shannon, Ace, and Chao indices, compared to the NILM group.

**Conclusion:**

These findings suggest that HPV infection and its associated pathological conditions are closely linked to alterations in the vaginal microbiome. This underscores the need for further research to unravel the intricate relationship between HPV genotype infections and vaginal microbiota, which could pave the way for new diagnostic and therapeutic approaches.

## Introduction

Human papillomavirus (HPV) is a dsDNA, non-enveloped virus that commonly infects the genital tract. It is a major causative agent of cervical intraepithelial neoplasia (CIN) which has the potential to progress to cervical cancer (Gheit, 2019). Cervical carcinoma remains a significant public health concern, particularly in underdeveloped regions, such as Africa where mortality rates are disproportionately high. Cervical cancer ranked among the top three cancers affecting women aged 45-yearsin 146 out of 185 assessed countries. Eswatini reports the highest incidence, with a rate of approximately 6.5%. Notably, China and India together account for over one-third of the global cervical cancer burden, with 106,000 and 97,000 cases reported annually, respectively. Their mortality rates are equally alarming, with approximately 48,000 deaths in China and 60,000 in India each year (Arbyn et al., 2020). The global average age for cervical cancer diagnosis is 53 years, while the average age at death from the disease is59 years (Arbyn et al., 2020). HPV is commonly transmitted to women during sexual intercourse. However, most infections resolve within 2.5 years, depending on factors such as the HPV genotype, viral load, and the host’s immune response (Kombe Kombe et al., 2020). Women who achieve viral clearance and maintain normal cervical cytology are at a significantly lower risk of developing cervical intraepithelial neoplasia (CIN) (Gilham et al., 2019; Borgogna et al., 2020). In the case of HPV latency and reactivation, the persistent infection may lead to CINI, CINII, CINIII, or cervical cancer (Gilham et al., 2019).

HPV genotypes have been grouped into high-risk (HR-HPVs) and low-risk (LR-HPVs) depending on their carcinogenicity. For example, HR-HPVs are responsible for persistent cervical infections, lesions, cervical cancers, oropharyngeal cancers, and anogenital cancers, whereas LR-HPVs cause anogenital and cutaneous warts in infected individuals (Kombe Kombe et al., 2020; Usyk et al., 2020). In the case of persistent HPV infections, the viral genome is integrated into the host genome to cause cervical cancer (Usyk et al., 2020). Previous findings show that HPV16 and 18 are the most frequent genotypes responsible for approximately 75% of cervical cancers globally. While HPV31, 33, 35, 45, 52, and 58 vary among different countries and regions, causing 20% of cervical cancers (de Sanjose et al., 2010; Li et al., 2011). HPV31 and 33 are more common in Europe and America, while genotypes 35,45 are more common in Africa, and HPV 58 and 52 in Asia (de Sanjose et al., 2010). Our recent study reported that HPV16, 52, 18, 58, and 53 were the most prevalent genotypes responsible for cervical cancer and other gynecological-related problems in different regions of Yunnan, China (Baloch et al., 2015) (unpublished data). Therefore, regional data on HPV prevalence and its various genotype distributions are important for estimating the impact of vaccines on cervical cancer. In addition to the HPV genotypes, risk factors, such as sexual practices, smoking, contraceptives, etc., were reportedly linked with the HPV infection and its progression to cervical carcinomas (Kombe Kombe et al., 2020).

The vaginal microbiota is a complex community of microorganisms, including bacteria, fungi, protozoa, and viruses. Under normal conditions, these microorganisms maintain a delicate balance, both among themselves and with the host, playing a vital role in maintaining vaginal health (Avitabile et al., 2024). However, when this balance is disrupted, it can lead to the development of various gynecological conditions. In healthy women, the vaginal microbiota is primarily dominated by Lactobacillus species, including L. crispatus, L. gasseri, L. iners, and L. jensenii (Smith and Ravel, 2017). These bacteria produce bacteriocins, hydrogen peroxide, and lactic acid, which help inhibit the growth of pathogenic microbes, regulate the immune response, and enhance the vagina’s resistance to infections (Aldunate et al., 2015). Research has found that dysbiosis of the vaginal microbiome in HPV-infected women is linked to several gynecological disorders, with bacterial imbalances playing a key role. One study revealed a significant relationship between HPV infection and variations in the vaginal microbiota’s composition (Kombe Kombe et al., 2020).

The vaginal microbiota abundance characterized five microbial community state types (CST) in asymptomatic female cases using 16S rRNA gene sequencing (Ravel et al., 2011). *Lactobacillus* dominated four (CST-I, II, III, and V). A lower abundance of *Lactobacillus* and higher levels of *Gardnerella*, *Mobiluncus*, *Mycoplasma*, and *Prevotella* were characterized in CST-IV cases (Ravel et al., 2011). However, the vaginal microbiome’s function and pathophysiological role in HPV infections are largely unknown. It has been suggested that the vaginal microbiome influences HR-HPV persistence infection and the occurrence of CINI, CINII, CINIII, and cervical cancers (Brusselaers et al., 2019). Despite growing evidence, the role of diverse vaginal microbial communities in HPV genotype infections, CIN I, CIN II, CIN III, and cervical cancer remains insufficiently explored. Therefore, this study aimed to investigate whether HPV genotype infections and associated cervical conditions influence the composition and diversity of vaginal microbiome communities in women from Yunnan Province, China.

## Methods

### Study design

In this study, 69 women, including 16 healthy controls, were recruited from the Out-Patient Department of the First People’s Hospital of Yunnan Province between December 2018 and September 2020. Participants were excluded if they met any of the following criteria: vaginal washing within 24 hours prior to sampling, use of vaginal medications within two weeks, sexual activity within three days, oral antibiotic use within two weeks, or a history of cervical therapy. A standardized questionnaire was administered to collect detailed participant information, including ethnicity, education level, age, marital status, smoking and drinking habits, sexual activity, occupation, and other relevant factors.

### Ethical statement

This study was approved by the Ethics Committee of the Faculty of Life Science and Technology at Kunming University of Science and Technology, as well as the Center for Disease Control and Prevention (CDC) in Yunnan Province, China. Written informed consent was obtained from all participants, and all experiments were conducted in accordance with the regulations and guidelines of the Ethics Committee.

### Sample collection

For sample collection, a cotton swab (Santai, Jiangsu, China) was inserted 2−3 cm into the vagina, gently rotated for 15−20 seconds, and then placed into the 1.5 ml tube with 1.0 ml 0.9% physiological saline. The swab samples containing microbial content were transported to the laboratory in 30 minutes on ice. There, each specimen was centrifuged at 10000× g for 10 min at room temperature. After centrifugation, the supernatant was discarded, and the bacterial sediment was preserved at −80°C for further analysis.

### Histopathological analysis

During colposcopy, the cervix was divided into four quadrants, and each was carefully examined individually. Biopsies were taken from areas with visible abnormalities, while a random biopsy was collected from the squamocolumnar junction in quadrants that appeared normal. Endocervical curettage was also performed. Cervical biopsies were obtained using standard 2 mm POI biopsy forceps, which facilitate quick healing and reduce patient discomfort. Histological slides were reviewed by two senior pathologists from Yunnan First People’s Hospital. Diagnoses of cervical intraepithelial neoplasia (CIN I–III) and cervical cancer were made based on the World Health Organization’s classification system.

### HPV genotyping

HPV genomic DNA was extracted from each sample using the TIANamp DNA Extraction Kit (TIANgen Biotech, Co., Hong Kong), following manufacturer instructions. The extracted DNA was amplified through PCR using consensus primers (MY09/11) targeting the HPV L1 region, employing the GenoArray Test Kit (Hybribio, Chaozhou, China). The HPV L1 consensus primer was used to amplify twenty-three HPV genotypes, including thirteen HR-HPVs (HPV16, 18, 31, 33, 35, 39, 45, 51, 52, 56, 58, 59, 68, 53, 66, and 81), and seven LR-HPVs (HPV6, 11, 42, 43, 44, and 61), following manufacturer protocol. Positive controls consisted of PCR-amplified DNA from the HeLa and Caski cell lines, while negative controls included PCR mixture without sample DNA.

### 16S rRNA gene sequencing and analysis

Genomic DNA from each vaginal sample was extracted using the TIANamp DNA Extraction Kit (TIANgen Biotech, Co., Hong Kong) according to the manufacturer’s instructions. After extraction and purification, DNA concentration was quantified with the Qubit v2.0 Fluorometer (Thermo Fisher Sci., USA), and its integrity was confirmed via agarose gel electrophoresis. The KAPA HTP/LTP Library Preparation Kits (Kapa Bio-systems, USA) were used to construct the DNA library for sequencing on the Agilent 2100 (Agilent, US) sequencer. The libraries from each sample were then sequenced on the MiSeq Illumina benchtop System (Illumina, California, USA) at Shanghai Majorbio Bio-Pharm Technology, China. The 16S rRNA gene V4 region was amplified using consensus primers, 5′CCTACGGGNGGCWGCAG3′, and 5′GACTACHVGGGTATCTAATCC3′, targeting the vaginal microbiome. Raw reads were processed by trimming low-quality bases, removing sequences with unknown N bases, adapters, and bases with Phred scores below 20. The resulting paired-end clean reads were aligned against the known microbial genomes in the NCBI using SOAP aligner v2.21 (Li et al., 2008). The sequences mapped to the host genome were abandoned while the subsequent sequences were subjected to downstream analysis.

### Taxonomic profiling

Taxonomic profiling was conducted at the family, genus, and species levels. After *de novo* assembling into contigs, all clean reads were clustered into operational taxonomic units (OTUs) with a 97% similarity threshold. Mothur v1.30.1 was used to determine alpha microbial diversity, while beta diversity was determined using Quantitative Insights Into Microbial Ecology (QIIME). Microbial diversity indicators, such as Chao and ACE, were calculated using the Calypso tools (http://www.mothur.org/wiki/Chao; http://www.mothur.org/wiki/Ace). Bacterial abundance was assessed using Shannon and Simpson’s indices (http://www.mothur.org/wiki/Shann; http://www.mothur.org/wiki/Simps). Principal coordinates analysis (PCoA) was performed using Bray-Curtis dissimilarity matrices. Linear discriminant analysis (LDA) with effect size (LEfSe) was carried out using the LEfSe program to identify significant differences in microbial communities (Segata et al., 2011). Moreover, Tax4Fun2, a powerful open-source R package, predicts the functional capabilities of microbiomes. Further, we compared the coverage of the pipelines in terms of reads classified and mapped to KEGG and COG databases.

### Statistical analysis

The significance of each taxon (phylum, family, genus, and species, between women with HPV+, HPV genotype, CINI, CINII, CINIII, cervical cancer, and NILM) was then determined using a non-parametric unpaired two-sample Wilcoxon test. P-values were FDR-corrected using the Benjamini-Hochberg method. Enriched features of each group with an adjusted *P* < 0.05 were identified based on the Wilcox rank-sum value. KEGG, COG, and KO were used to determine the organism’s features among the HPV+, CINI, CINII, CINIII, cervical cancers, and NILM. The LEfSe analysis explained the differences between the microbiomes (White et al., 2009). To compare the relationships between HPV+, ethnicity, married status, age, smoking, drinking, and the number of sexual partners, Fisher’s exact test was used.

## Results

### Demographic characteristics

The socio-demographic characteristics of the 69 participants are summarized in **Table 1**. The mean age ± SD of women diagnosed with HPV+, CIN I, CIN II, CIN III, and cervical cancer was 37.14 ± 10.02, 37.17 ± 5.08, 42.93 ± 9.28, and 46.5 ± 4.50 years, respectively. Among the ethnic groups, the majority of participants were of Han ethnicity, comprising 81.2% of HPV+ cases, 71.4% of CIN I, 83.3% of CIN II, and 80% of CIN III cases. In contrast, other ethnicities accounted for 18.8% of HPV+ cases, 28.6% of CIN I, 16.7% of CIN II, and 20% of CIN III cases. Notably, no cervical cancer cases were reported among women from non-Han ethnic groups. The educational background of HPV+ participants revealed that 18.8% had completed primary school or below, 50% had a middle school education, and 31.2% had attained a college education or higher.

**Table 1:** Socio−demographic characteristics of the 69 participant.

Parameter	HPV+ (n=16)	CINI (n=14)	CINII (n=6)	CINIII (n=15)	Cervical cancer (n=2)	NILM (n=16)
Age						
Mean age ± SD	36.13 ± 9.40	37.14 ± 10.02	37.17 ± 5.08	42.93 ± 9.28	46.5 ± 4.50	35.75 ± 7.94
Age range (years)	22 ~ 55	22 ~ 53	29 ~ 45	28 ~ 59	42 ~ 51	23 ~ 49
Ethnic, n (%)						
Han	13 (81.2)	10 (71.4)	5 (83.3)	12 (80)	2 (100)	11 (68.8)
Others	3 (18.8)	4 (28.6)	1 (16.7)	3 (20)	0 (0)	5 (31.2)
Education level						
Primary/below	3 (18.8)	2 (14.3)	2 (33.3)	3 (20)	1 (50)	4 (25)
Middle	8 (50)	8 (57.1)	3 (50)	6 (40)	1 (50)	7 (43.8)
College/above	5 (31.2)	4 (28.6)	1 (16.7)	6 (40)	0 (0)	5 (31.2)
Marriage status						
Married	14 (87.5)	12 (85.7)	6 (100)	14 (93.3)	2 (100)	16 (100)
Unmarried	2 (12.5)	1 (7.1)	0 (0)	1 (6.7)	0 (0)	0 (0)
Smoking						
Yes	0 (0)	0 (0)	0 (0)	1 (6.7)	0 (0)	1 (6.2)
No	16 (100)	14 (100)	6 (100)	14 (93.3)	2 (100)	15 (93.8)
Drinking						
Yes	0 (0)	1 (7.1)	0 (0)	2 (13.3)	0 (0)	1 (6.2)
No	16 (100)	13 (92.9)	6 (100)	13 (86.7)	2 (100)	15 (93.8)
Sexual partner						
1	13 (81.3)	11 (78.6)	4 (66.7)	12 (80)	2 (100)	15 (93.8)
2 or more	3 (18.7)	3 (21.4)	2 (33.3)	3 (20)	0 (0)	1 (6.2)

All the data is represented as mean ± standard deviation (± SD) and the p-value < 0.05 was considered significant.

### Distribution of HR-HPV and LR-HPV

The overall high-risk HPV (HR-HPV) infection rates were 88.46% in HPV+ cases, 28.99% in CIN I, 13.04% in CIN II, and 24.64% in CIN III. In contrast, low-risk HPV (LR-HPV) infection rates were 7.25% in CIN I, 4.35% in CIN II, and 4.35% in CIN III. Notably, no LR-HPV infections were detected in cervical cancer cases (**Table 2**). Among the HR-HPV types, HPV52 showed the highest prevalence at 23.08%, followed by HPV16, which was found in 20% of CIN I cases and 25% of CIN III cases. In cervical cancer cases, two HR-HPV genotypes—HPV16 and HPV52—were identified, while no LR-HPV types were reported.

**Table 2:** Distribution of HR-HPV and LR-HPV genotypes in women diagnosed with various CIN, and cervical cancer.

HPV type	HPV+ 26 (%)	CINI, 25 (%)	CINII, 12 (%)	CINIII, 20 (%)	Cervical cancer, 2 (%)	Overall 85 (%)
HR-HPV						
HPV-16	3 (11.54)	5 (20)	1 (8.33)	5 (25)	1 (50)	15
HPV-18	1 (3.85)	1 (4)	0 (0)	1 (5)	0 (0)	3
HPV-31	0 (0)	0 (0)	0 (0)	1 (5)	0 (0)	1
HPV-33	3 (11.54)	2 (8)	1 (8.33)	4 (20)	0 (0)	10
HPV-34	1 (3.85)	0 (0)	0 (0)	0 (0)	0 (0)	1
HPV-35	1 (3.85)	2 (8)	0 (0)	0 (0)	0 (0)	3
HPV-51	1 (3.85)	3 (12)	1 (8.33)	0 (0)	0 (0)	5
HPV-52	6 (23.08)	2 (8)	1 (8.33)	2 (10)	1 (50)	12
HPV-53	2 (7.69)	2 (8)	0 (0)	0 (0)	0 (0)	4
HPV-58	1 (3.85)	1 (4)	2 (16.66)	4 (20)	0 (0)	8
HPV-59	1 (3.85)	1 (4)	1 (8.33)	0 (0)	0 (0)	3
HPV-66	1 (3.85)	0 (0)	2 (16.66)	0 (0)	0 (0)	3
HPV-68	2 (7.69)	1 (4)	0 (0)	0 (0)	0 (0)	3
LR-HPV						
HPV-6	0 (0)	1 (4)	0 (0)	0 (0)	0 (0)	1
HPV-11	0 (0)	0 (0)	1 (8.33)	0 (0)	0 (0)	1
HPV-43	1 (3.85)	0 (0)	0 (0)	0 (0)	0 (0)	1
HPV-55	0 (0)	0 (0)	1 (8.33)	0 (0)	0 (0)	1
HPV-61	1 (3.85)	0 (0)	1 (8.33)	0 (0)	0 (0)	2
HPV-72	0 (0)	0 (0)	0 (0)	2 (10)	0 (0)	2
HPV-81	1 (3.85)	1 (4)	0 (0)	1 (5)	0 (0)	3
HPV-82	0 (0)	1 (4)	0 (0)	0 (0)	0 (0)	1
HPV-83	0 (0)	1 (4)	0 (0)	0 (0)	0 (0)	1
HPV-84	0 (0)	1 (4)	0 (0)	0 (0)	0 (0)	1

HR-HPV, high-risk HPV genotypes; LR-HPV, low-risk HPV genotypes.

In this study, the overall HR-HPV infection rate was 83.53%, while 16.47% of infections were attributed to LR-HPV. Among the HR-HPV genotypes, HPV16 was the most prevalent, detected in 15 cases across women with HPV+, CIN I, CIN II, CIN III, and cervical cancer. This was followed by HPV52 and HPV33, which were identified in twelve and ten cases, respectively. Specifically, six cases of HR-HPV52 were found in the HPV+ group, while five cases of HPV16 were detected in both CIN I and CIN III groups.

### Vaginal microbiota

**Figure 1:**
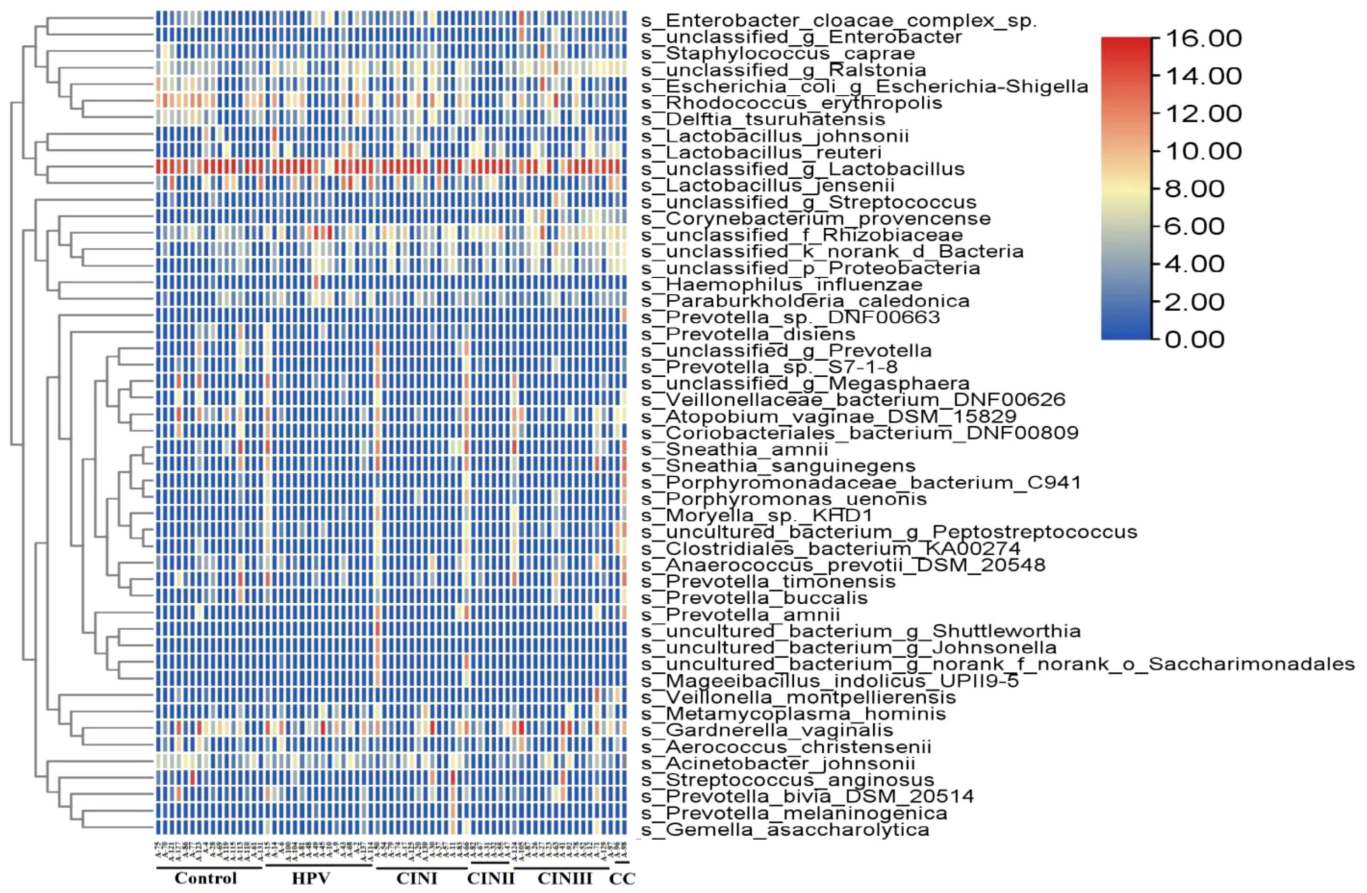
Heatmap shows diverse microbial communities in women with HPV+, CINI, CINII, CINIII, cervical cancers, and NILM (control). The color bar from red to blue represents the relative abundance of bacterial species from high to low.

**Figure 2:**
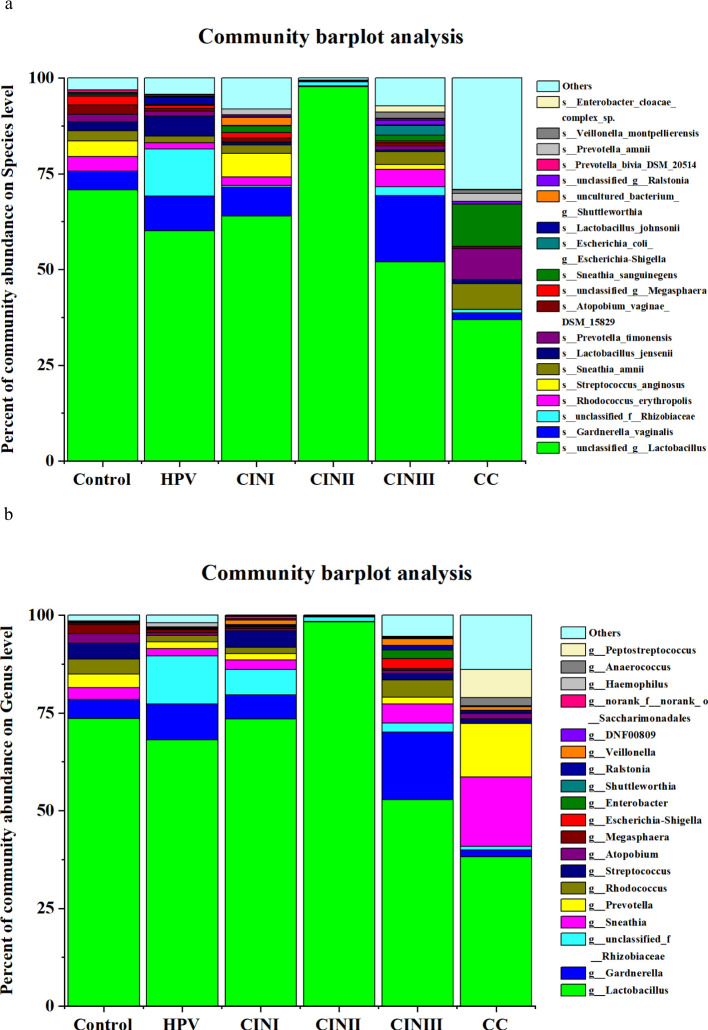
The vaginal microbiota in women with HPV+, CINI, CINII, CINIII, cervical cancers, and NILM (control) at species **(A)** and genus levels **(B)**. (the participants received no treatment till sample collection).

A 16S rRNA-targeted metagenomics analysis was performed to determine bacterial abundance of each participant. A heatmap diagram depicts different bacterial communities in women with HPV+, CINI, CINII, CINIII, cervical cancers, and control. According to the heatmap diagram, the bacteria with the highest abundance belonged to the species unclassified of the genus *Lactobacillus*. The bar color represents the richness of bacterial communities ranging from bottom to top (**Figure 1**). The details of the bacterial abundance at species and genus levels are shown in **Figure 2**.

**Figure 3:**
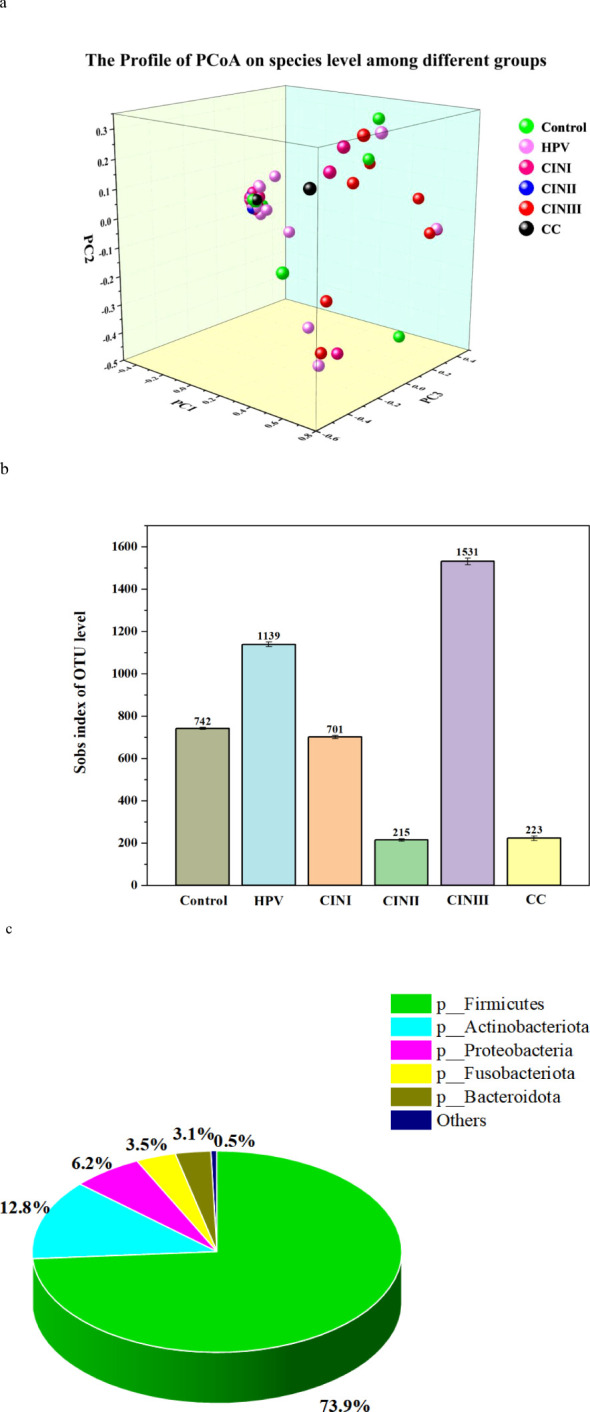
PCoA profile, Sobs index of OUT level, and taxonomic biomarkers LEfSe analysis of the cohort associated with HPV+, CINI, CINII, CINIII, cervical cancers, and NILM (control). **(A)** PCoA profile of the cohort associated with HPV infection status. Each dot presented a sample, and the color was according to each group, i.e., HPV+, CINI, CINII, CINIII, cervical cancers, and NILM (control). **(B)** The alpha-diversity (Sobs index of OUT level) between HPV+, CINI, CINII, CINIII, cervical cancers, and NILM (control). **(C)** Taxonomic biomarkers: the LEfSe analysis among HPV+, CINI, CINII, CINIII, cervical cancers, and NILM (control).

The details of the α-diversity indicators (i.e., Sobs, Shannon, Simpson, Ace, Chao, Coverage, Shannon, and Simpson) of the women with HPV+, CINI, CINII, CINIII, cervical cancers, and NILM at the species level are shown in **Supplementary Table S1**. In HPV+ cases, the Sobs index was significantly higher (62.63 ± 36.49) than those in CINI (42.64 ± 23.62), CINII (28.17 ± 13.41), and the control (40.25 ± 19.39) (*p* = 0.001), indicating diverse bacterial communities. In contrast, the Sobs index was higher (70.80 ± 41.08) in CINIII and cervical cancer (76.50 ± 13.50) than in the rest of the groups. The Shannon index (0.68 ± 0.51) in HPV+ women was significantly higher than CINII (0.13 ± 0.07) and control (0.67 ± 0.56), but less than CINI (0.72 ± 0.86), CINIII (0.88 ± 0.71), and cervical cancer (1.82 ± 0.67) (*p* = <0.001), suggesting increased vaginal microbiota in HPV+ cases and decreased in NILM. In brief, diversity indicators, Ace, Chao, and Coverage, showed significantly different (*p* = <0.001) bacterial communities among the women with different pathological conditions. Moreover, the details of the alpha-microbial diversity indicators at the genus level are shown in **Supplementary Table S2**. Furthermore, the bacterial β-diversity profile (PCoA) among HPV+, CINs, cervical cancer cases, and NILM is shown in **Figure 3A**.

Taxon identity and abundance were also established based on OTU levels. Identical OTU, species abundance, and sobs index was found in the vaginal microbiome of women with HPV+, CINI, CINII, CINIII, cervical cancer, and NILM (**Figure 3B**). The highest identical bacterial abundance was found in women with CINIII (1531), followed by HPV+ (1139), CINI (701), cervical cancer (223), and CINII (215).

### Understanding microbial alteration in different groups

To investigate the correlation in vaginal microbiota in HPV+, CINI, CINII, CINIII, cervical cancers, and NILM, LEfSe analysis was performed (**Figure 3C**). The LDA scores showed that unclassified species within the genus *Lactobacillus* were the most dominant bacteria in CINI, whereas uncultured species within the genus *Paenochrobactrum* were distinct among all groups. In the HPV+, *Lactobacillus reuteri* was predominant, followed by Burkholderia caballeronia paraburkholderia, *Tepidiphilus*, and unclassified species in the genus Norank. Interestingly, *Lactobacillus fermentum* was the only species present in the CINI. In the CINII, only two taxa were identified: unclassified species within the genus *Lactobacillus* and *Capnocytophaga*. For CINIII, the family *Burkholderiaceae* and unclassified species within the genus *Ralstonia* were the most common, followed by the family *Corynebacteriaceae* and the genus *Corynebacterium*.

## Discussion

The vaginal microbiome has garnered growing interest for its potential influence on the genital tract environment, its critical role in female reproductive health, and its association with HPV infection (Anahtar et al., 2018). HPV infection is widespread, with its prevalence and impact varying significantly across and within regions. These differences are influenced by factors such as age, gender, geographical location, lifestyle, and socio-economic conditions (LeConte et al., 2018). Aside from cervical cancer and its precursors, growing evidence suggests that HPV infection plays a crucial role in diseases of the lower genital tract, particularly in the development of vaginal and vulvar precancerous conditions (Bogani et al., 2023). In our study, the mean age (46.5 ± 4.50 years), ranging from 42–51 years of participants for cervical cancer diagnosis, was lower when compared to other life-threatening cancers (Yang et al., 2004).

The management of HPV-related lesions continues to be a global challenge, with significant implications for patient prognosis. Recent studies, such as the work by Golia D’Augè et al. (2024), highlight the growing importance of a personalized, tailored approach to managing HPV-related cervical lesions. Advances in our understanding of HPV pathogenesis, combined with improved diagnostic tools, now allow for more precise management strategies. These strategies consider factors such as lesion severity, HPV type, vaccination status, and the patient’s immune profile, all of which contribute to the optimal approach for treatment. Tailored management not only improves the prognosis of patients with HPV-related lesions but also has the potential to prevent the progression of lesions to cervical cancer. In light of these developments, our study aims to contribute to the ongoing conversation by further elucidating the role of the vaginal microbiota in influencing the persistence and progression of HPV infections.

According to the predictions, the dynamics of HPV transmission and the consequences of HPV infection in developing and underdeveloped countries would be similar to those in developed countries (WHO, 2022). It is worth mentioning that there was only one CINI woman with LR-HPV6 infection. According to our findings, HPV16 genotype infection was much less than that reported in Beijing in 2014 and 2015 (Shen et al., 2018). A study identified 396 HPV genotypes in infected people using metagenomic sequencing (Bzhalava et al., 2014). Among them, 14 genotypes were HR-HPV due to their oncogenic potential, with HPV16 being the most prevalent genotype, in line with previous findings (Crow, 2012).

In this cohort, we explored the diverse vaginal microbiome in women with HPV+, CINI, CINII, CINIII, cervical cancers, and NILM through high-throughput sequencing. Our findings were consistent with another study that found *Lactobacillus* to be the dominant genus (Anahtar et al., 2018), creating an acidic environment via lactic acid production to protect women from opportunistic pathogens and HPV infection. The increased *Gardnerella vaginalis*, *Rhizobiaceae*, and decreased *Peptostreptococcus* and *Enterobacter cloacae complex* were closely associated with HPV-associated gynecological disorders. The vaginal microbiome is primarily dominated by certain *Lactobacillus* species like *L. crispatus*, *L. gasseri*, *L. jensenii*, and *L. iners* (Borgogna et al., 2020), which maintain an acidic environment through lactic acid production. In contrast, Lee and Collogue reported a lower proportion of *Lactobacillus* in HPV-infected women (Lee et al., 2013). Studies also reported that *Lactobacillus* could inhibit the growth of other opportunistic pathogens by protecting the lower reproductive tract from infection (Martin and Marrazzo, 2016; Nunn and Forney, 2016). In consistent with our study, *Lactobacillus* abundance was lower in HPV16-infected individuals than in NILM. *Lactobacillus* normally adheres to the genital tract epithelial cells and secretes lactic acid via glycogen decomposition, maintaining a mild acidic vaginal environment to prevent pathogenic microbes from colonizing. These bacteria also produce antimicrobial secondary metabolites such as bacteriocins to prevent and inhibit pathogenic microbes from colonization, thereby maintaining a normal environment (Boris and Barbés, 2000; Tachedjian et al., 2017). In contrast, reduced *Lactobacillus* promotes opportunistic bacterial growth such as *Fusobacterium*, *Gardnerella*, *Mobiluncus*, *Parvimonas*, *Peptostreptococcus*, and *Prevotella* in HPV16-infected women. It has been shown that *Gardnerella* may produce virulence factors, adhesion, and cytotoxin (Nowak et al., 2018) that inhibit the growth of pathogens, further suggesting its potential role in vaginal dysbiosis.

Generally, less diversity in microbial communities signifies good health (Turnbaugh et al., 2007). The sob, Ace, and Chao indexes of α-diversity of the vaginal microbiome at the species and genus levels showed an increasing trend among women with HPV+, CINI, CINII, CINIII, and cervical cancers and NILM. The sobs, Shannon, Ace, and Chao coverage α-diversity identified at the species level among the women with various gynecological disorders and NILM significantly increased estimated OTUs compared with those in NILM. On the other hand, the Simpson, Shannoneven, and Simpsoneven indexes of α-microbial diversity at the species level showed no significant difference (*p* = 0.094; *p* = 0.096; *p* = 0.106). In contrast, we did not observe any significant difference in Shannon, Simpson, Shannoneven, and Simpsoneven indicators at the genus level. We further analyzed β-diversity to evaluate species complexity among HPV+, CINI, CINII, CINIII, cervical cancers, and NILM. Thus, our findings suggest that maintaining a stable vaginal microbiome may potentially prevent HPV infection. Globally, managing HPV-related lesions remains a significant challenge. In the future, modulating the vaginal microbiome could offer a promising strategy for this patient group, providing a novel approach to overcoming current therapeutic limitations.

## Conclusions

This study revealed significant alterations in the vaginal microbiota of women with HPV infection, CIN I, CIN II, CIN III, and cervical cancer. Specifically, an increased presence of Lactobacillus and a reduced abundance of the Enterobacter cloacae complex, Peptostreptococcus, and other microorganisms were observed. These findings suggest that disruptions in the vaginal microbiota may contribute to persistent HPV infection, progression to cervical intraepithelial neoplasia (CIN), and the development of cervical cancer. Moreover, infections with multiple HPV genotypes appear to influence the composition of the vaginal microbiome, notably affecting organisms such as Gardnerella vaginalis and members of the Rhizobiaceae family. Further research is essential to elucidate the complex interplay between persistent HPV infection and vaginal microbiota diversity, paving the way for the discovery of affordable therapeutic interventions in the future.

## Data Availability

The data presented in the study are deposited in the https://www.ncbi.nlm.nih.gov/, accession number PRJNA799456.
