# Supplementary material for: Vaginal microbiome distinction in women with HPV+, cervical intraepithelial neoplasia, and cervical cancer, a retrospective study
Source: Front Cell Infect Microbiol. 2025 Jan 17;14:1483544. doi: 10.3389/fcimb.2024.1483544 (PMC11782028; doi:10.3389/fcimb.2024.1483544)
Supplement: Supplementary file 1 [file DataSheet1.pdf]

**Table S1. The α-diversity of the vaginal microbial community at the species level.** All the data is represented as mean ± standard deviation (± SD) and the *p-value* < 0.05 was considered significant.

| **Diversity indicators** | **HPV+** | **CINI** | **CINII** | **CINIII** | **Cervical cancer** | **Control** | ***P*-value** |
|---|---|---|---|---|---|---|---|
| Sobs | 62.63 ± 36.49 | 42.64 ± 23.62 | 28.17 ± 13.41 | 88.27 ± 52.43 | 93.00 ± 20.00 | 40.25 ± 19.39 | 0.001 |
| Shannon | 0.68 ± 0.51 | 0.72 ± 0.86 | 0.13 ± 0.07 | 0.88 ± 0.71 | 1.82 ± 0.67 | 0.67 ± 0.56 | <0.001 |
| Simpson | 0.71 ± 0.23 | 0.74 ± 0.29 | 0.96 ± 0.03 | 0.65 ± 0.29 | 0.35 ± 0.24 | 0.71 ± 0.25 | 0.094 |
| Ace | 87.26 ± 43.20 | 56.26 ± 30.97 | 46.28 ± 13.28 | 150.80 ± 76.60 | 155.98 ± 11.85 | 52.39 ± 27.54 | <0.001 |
| Chao | 79.29 ± 40.32 | 53.51 ± 25.59 | 37.83 ± 18.20 | 127.20 ± 59.51 | 128.18 ± 5.04 | 47.63 ± 23.31 | <0.001 |
| Coverage | 0.9996 ± 0.0003 | 0.9997 ± 0.0001 | 0.9997 ± 0.0001 | 0.9993 ± 0.0004 | 0.9993 ± 0.0001 | 1.00 ± 0.00 | <0.001 |
| Shannoneven | 0.16 ± 0.12 | 0.18 ± 0.19 | 0.04 ± 0.02 | 0.19 ± 0.15 | 0.41 ± 0.17 | 0.18 ± 0.15 | 0.096 |
| Simpsoneven | 0.03 ± 0.02 | 0.06 ± 0.04 | 0.05 ± 0.03 | 0.03 ± 0.02 | 0.07 ± 0.05 | 0.05 ± 0.04 | 0.106 |

**Table S2. The α-diversity of the vaginal microbial community at the Genera level.** All the data is represented as mean ± standard deviation (± SD) and the *p-value* < 0.05 was considered significant.

| **Diversity indicators** | **HPV+** | **CINI** | **CINII** | **CINIII** | **Cervical cancer** | **Control** | ***P*-value** |
|---|---|---|---|---|---|---|---|
| Sobs | 51.94 ± 30.09 | 35.21 ± 17.65 | 21.83 ± 10.54 | 70.80 ± 41.08 | 76.50 ± 13.50 | 33.63 ± 14.43 | 0.001 |
| Shannon | 0.48 ± 0.45 | 0.63 ± 0.76 | 0.09 ± 0.08 | 0.82 ± 0.70 | 1.50 ± 0.47 | 0.54 ± 0.53 | 0.063 |
| Simpson | 0.80 ± 0.21 | 0.76 ± 0.28 | 0.97 ± 0.04 | 0.66 ± 0.30 | 0.42 ± 0.21 | 0.77 ± 0.25 | 0.093 |
| Ace | 70.85 ± 36.75 | 43.08 ± 20.33 | 36.26 ± 19.24 | 114.87 ± 58.88 | 130.93 ± 21.48 | 42.59 ± 27.76 | <0.001 |
| Chao | 63.72 ± 34.15 | 42.26 ± 18.59 | 28.74 ± 14.42 | 99.36 ± 45.93 | 105.15 ± 0.71 | 39.74 ± 18.34 | <0.001 |
| Coverage | 0.9997 ± 0.00 | 0.9998 ± 0.00 | 0.9998 ± 0.00 | 0.9994 ± 0.00 | 0.9994 ± 0.00 | 1.00 ± 0.00 | <0.001 |
| Shannoneven | 0.12 ± 0.11 | 0.17 ± 0.18 | 0.03 ± 0.02 | 0.19 ± 0.15 | 0.35 ± 0.12 | 0.16 ± 0.15 | 0.122 |
| Simpsoneven | 0.04 ± 0.03 | 0.06 ± 0.04 | 0.07 ± 0.04 | 0.03 ± 0.02 | 0.05 ± 0.03 | 0.06 ± 0.04 | 0.113 |
